# Direct interaction studies between *Aspergillus fumigatus* and human immune cells; what have we learned about pathogenicity and host immunity?

**DOI:** 10.3389/fmicb.2012.00413

**Published:** 2012-12-03

**Authors:** Charles O. Morton, Maria Bouzani, Juergen Loeffler, Thomas R. Rogers

**Affiliations:** ^1^School of Science and Health, University of Western SydneyCampbelltown, NSW, Australia; ^2^Medizinische Klinik und Poliklinik II, Universitätsklinikum WürzburgWürzburg, Germany; ^3^Department of Clinical Microbiology, Trinity College Dublin, Sir Patrick Dun Research Laboratory, St James’s HospitalDublin, Ireland

**Keywords:** *Aspergillus*, innate immunity and responses, host–pathogen interactions, aspergillosis, cellular immunity

## Abstract

Invasive aspergillosis is a significant threat to health and is a major cause of mortality in immunocompromised individuals. Understanding the interaction between the fungus and the immune system is important in determining how the immunocompetent host remains disease free. Several studies examining the direct interaction between *Aspergillus fumigatus* and purified innate immune cells have been conducted to measure the responses of both the host cells and the pathogen. It has been revealed that innate immune cells have different modes of action ranging from effective fungal killing by neutrophils to the less aggressive response of dendritic cells. Natural killer cells do not phagocytose the fungus unlike the other innate immune cells mentioned but appear to mediate their antifungal effect through the release of gamma interferon. Transcriptional analysis of *A. fumigatus* interacting with these cells has indicated that it can adapt to the harsh microenvironment of the phagosome and produces toxins, ribotoxin and gliotoxin, that can induce cell death in the majority of innate immune cells. These data point toward potential novel antifungal treatments including the use of innate immune cells as antifungal vaccines.

## INTRODUCTION

The fungus *Aspergillus fumigatus* (teleomorph *Neosartorya fumigata*; O’Gorman et al., 2009) normally plays a role in the decay of plant organic matter in the environment (Gugnani, 2003). It is also an opportunistic pathogen that causes a spectrum of diseases in humans ranging from allergic reactions to life-threatening invasive disease in immunocompromised individuals (Neofytos et al., 2009). Invasive aspergillosis (IA) is the most severe disease caused by the fungus and is the leading cause of mycosis-related mortality in the immunocompromised (McCormick et al., 2010b).

Invasive aspergillosis is usually caused by the inhalation of asexual spores (conidia) into the alveoli; it is estimated that 200–300 conidia are inhaled daily (Latge, 1999). Conidia are covered in a layer of hydrophobin which is inert to the immune system (Aimanianda et al., 2009) and the presence of dihydroxynaphthalene (DHN)-melanin in the cell wall interferes with host endocytosis (Thywissen et al., 2011) leading to potential long-term survival of inhaled resting conidia. Proteomic analysis of resting conidia indicated the presence of enzymes that allow rapid adaptation to the environment in which the fungus germinates and the establishment of infection in the absence of competent antifungal defenses (Teutschbein et al., 2010). The cellular innate immune system usually provides protection against IA by neutralizing germinating conidia (Hasenberg et al., 2011).

**FIGURE 1:**
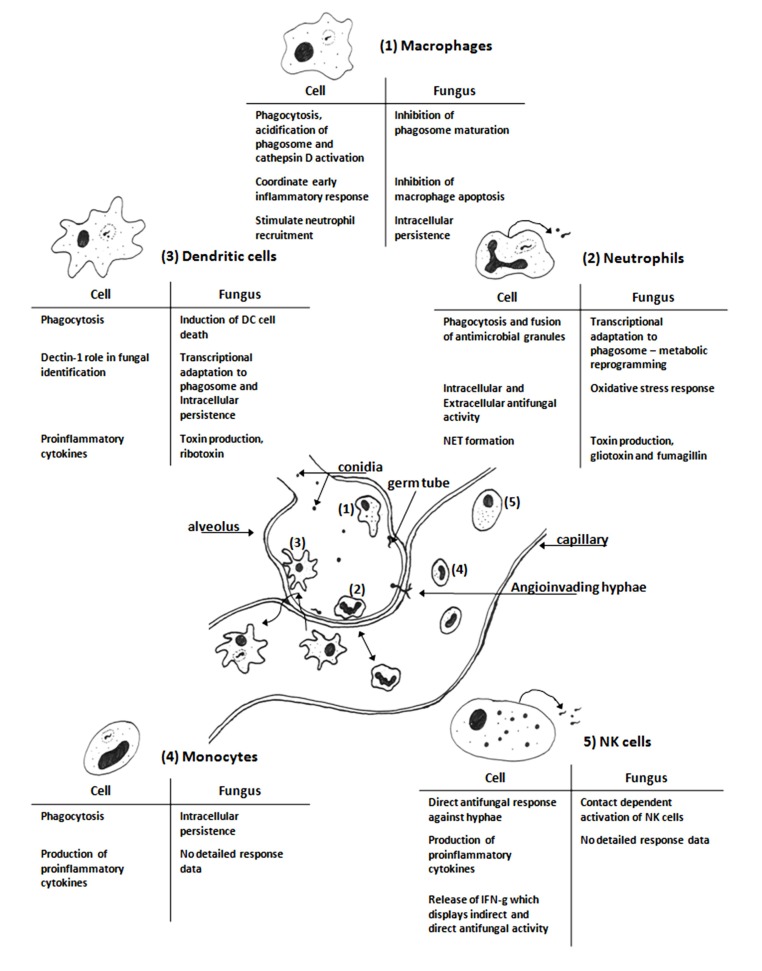
**Schematic diagram of cells of the innate immune system interacting with *Aspergillus fumigatus* in the vicinity of an alveolus.** The resident alveolar macrophages (1) are first to encounter the fungus and after phagocytosis they kill the ingested fungus and initiate the inflammatory response (Segal, 2007; Volling et al., 2007; Slesiona et al., 2012). Neutrophils (2) are recruited to the site of infection by chemoattractant cytokines, they are necessary for clearance of the fungal infection (Sugui et al., 2008; Brakhage et al., 2010; Fallon et al., 2010). Dendritic cells (3) actively scan the body for potential infectious agents; they phagocytose pathogens and drain to the lymph nodes where they can present antigens from the degraded pathogen to induce an adaptive immune response (Bozza et al., 2002; Mezger et al., 2008; Morton et al., 2011). Monocytes, progenitors for macrophages and some dendritic cell, (4) are present in blood; they can phagocytose fungal elements and release proinflammatory cytokines (Rodland et al., 2008; Loeffler et al., 2009). NK cells (5) are present in blood and can be attracted to sites of infection; they release antimicrobial molecules that can directly kill invading fungi (Park et al., 2009; Bouzani et al., 2011). The tables are summaries of the key features of the interactions between *A. fumigatus* with each immune cell based on the studies cited in each section. The figure is not to scale.

Immunity to fungal infection requires the concerted activity of the cells of the immune system (**Figure 1**). The importance of alveolar macrophages and neutrophils has been the subject of relatively extensive research and will not be the major focus of this review. We will primarily focus on dendritic cells (DC), monocytes, and the emerging role of natural killer (NK) cells in immunity to IA; examining how these analyses have added to the understanding of fungal virulence and immunity to IA.

## INTERACTIONS WITH INNATE IMMUNE CELLS

The first immune cells to interact with germinating fungal spores are the alveolar macrophages and neutrophils that are recruited to the site of infection. These cells recognize pathogen-associated molecular patterns (PAMPs), e.g., β-1,3-glucan, galactomannan, and chitin, that are exposed on the surface of germinating conidia or hyphae through pathogen-recognition receptors (PRR) such as Toll-like receptors (TLR; Netea et al., 2003) and Dectin-1 (Brown, 2006). It has been observed in murine models that neutrophils play an essential role in defense against germinating conidia (Mircescu et al., 2009; Ibrahim-Granet et al., 2010). Interference with the innate immune response is a primary factor in the initiation of IA; neutropenia is a primary risk factor for IA (De Pauw et al., 2008). However, increasing evidence suggests that neutrophils and macrophages act cooperatively to defend against infection (Silva, 2011).

## MACROPHAGES

The population of resident alveolar macrophages (AM) are thought to be the first immune cells to encounter *A. fumigatus* in the lung (**Figure 1**). The interaction between *A. fumigatus* and macrophages has provided an important model for the studying phagocytosis of the fungus. Conidia are phagocytosed by AM and killed following acidification of the phagolysosome and activation of antimicrobial enzymes (e.g., cathepsin D and chitinase; Ibrahim-Granet et al., 2003). Reactive oxygen species (ROS) are required to kill conidia but are not directly responsible for killing the fungus (Philippe et al., 2003; Cornish et al., 2008). AM along with direct antifungal activity also release cytokines to initiate an inflammatory response to fungal infection (Agarwal et al., 2007).

Fungal survival during interactions with AM is associated with fungal DHN-melanin inhibiting acidification of the phagolysosome (Thywissen et al., 2011). This promotes germination of the conidia and escape from the AM through hyphal growth (Slesiona et al., 2012). Another factor that is important for fungal survival during encounters with AM is the production of siderophores, deletion of genes encoding these iron scavenging molecules decreased fungal survival within the AM phagolysosome (Schrettl et al., 2010).

## NEUTROPHILS

Neutrophils are recruited to the site of infection by cytokines, primarily IL-8 in the lung, and are important players in the inflammatory response associated with the clearance of fungal infection (Bellocchio et al., 2004; Bonnett et al., 2006; Park and Mehrad, 2009). These cells recognize fungi through PRR–PAMP interactions and can phagocytose pathogenic microbes. Neutrophils are also capable of attacking hyphae through the production of neutrophil extracellular traps (NET) which are formed when neutrophils undergo autolysis releasing their DNA into the surrounding environment to impede the progress of infection (Bruns et al., 2010). NET formation is important in inhibition of hyphal growth and their formation can be induced by both conidia and hyphae (McCormick et al., 2010a). Interestingly, restoration of NET formation in neutrophils from a patient with chronic granulomatous disease (CGD) restored resistance to IA (Bianchi et al., 2011).

Transcriptional analysis of *A. fumigatus* that is directly interacting with neutrophils has been conducted and has revealed that there is a complex response by genes involved in oxidative stress and fatty acid catabolism (Sugui et al., 2008). Up-regulation of catalases (*cat1* and *cat2*), superoxide dismutase (Mn-SOD), and thioredoxin reductase are consistent with a reaction to oxidative stress caused by the generation of ROS within the phagolysosome in neutrophils.

There was a shift in the metabolism of the fungus to a state similar to that observed under glucose limitation indicating that phagocytosis by neutrophils creates nutrient limiting conditions (Sugui et al., 2008). Transcriptome analysis of *A. fumigatus* initiating infection in the murine lung revealed that nutrient limitation could be a cue for a trophic switch requiring the induction of putative virulence genes (McDonagh et al., 2008). This behavior has been seen in fungi developed as biological control agents where the absence of nutrients induced the expression of serine proteases that acted as virulence factors against invertebrates (St Leger et al., 1987; Segers et al., 1994). Up-regulation of secondary metabolite gene clusters including the gliotoxin gene cluster during infection of the murine lung may be important for the interaction with neutrophils since gliotoxin has been shown to inhibit phagocytosis and induce apoptosis of neutrophils (Comera et al., 2007).

## INTERACTIONS WITH ANTIGEN-PRESENTING CELLS

The inflammatory response initiated by AM and neutrophils also attracts antigen-presenting cells (APC) from the blood and surrounding tissues through the activity of cytokines (Burns and Thrasher, 2004) and alarmins, such as defensins (Oppenheim et al., 2007). The primary APC are DCs and monocytes. The APCs exist as a number of distinct subpopulations; monocyte subpopulations are CD14^+^CD16^–^ and CD14^+^CD16^+^ (Serbina et al., 2009). In humans 90% of circulating monocytes have CD14^+^ CD16^–^ markers, these can phagocytose conidia and inhibit germination but secrete small amounts of TNF-alpha. However, CD14^+^ CD 16^+^ monocytes, the remaining 10%, do not inhibit germination but secrete large amounts of TNF-alpha (Serbina et al., 2009). Monocytes are precursor cells for specific populations of DC and macrophages (Osugi et al., 2002).

The major subgroups of DC include myeloid DC (mDC), plasmacytoid DC (pDC), and monocyte-derived DC (moDC). Both mDC and pDC occur in low numbers within the bloodstream which is why moDC are frequently used in experiments since they can be generated in large numbers. It has been postulated that moDC in the body may represent an auxillary inflammatory pathway whereas mDC and pDC are the specialized surveillance subsets of DC (Osugi et al., 2002). APCs link the innate and adaptive arms of the immune response since the antigens induce a pathogen-specific T helper cell response (Bozza et al., 2002).

## DENDRITIC CELLS

Dendritic cells act as a surveillance system for the body; they can be found in the majority of tissues and the circulatory system in an immature state (Wuthrich et al., 2012). They act by sampling their microenvironment for potential microbial pathogens, uptake of a microbe leads to DC maturation and the presentation of microbial antigens in the lymph nodes activates specific T cells. These cells drive the adaptive immune system to produce a Th-1 or Th-2 type response; the inflammatory Th-1 response is associated with the clearance of fungal infection.

Whole genome analyses of the interaction between immature moDC, from healthy donors, and *A. fumigatus* have been conducted for both organisms. Analysis of the moDC transcriptome revealed that exposure to *A. fumigatus* induced a pro-inflammatory response and indicated the importance of Dectin-1 in fungal recognition. Among the genes that were up-regulated were *CCL20*, *IL1B*, *IL8*, and *PTX-3*. The expression of *SYK* and *IL2RA* were considered indicative of a role for DC in the activation of NK cells (Mezger et al., 2008). This expression pattern was confirmed by transcriptional analysis using a microarray of 120 immune-related genes which also showed the induction of a pro-inflammatory response of moDC to *A. fumigatus* (Morton et al., 2011).

Direct interaction between moDC and *A. fumigatus* leads to rapid phagocytosis of the fungal cells with phagocytosis of 48% of conidia after 2 h (Bozza et al., 2002) and 68% after 3 h (Morton et al., 2011). As mentioned with fungal survival in macrophages, germ tubes of *A. fumigatus* emerged from moDC after 6 h, which coincided with an increase in moDC cell death. Whole genome transcriptome analysis of *A. fumigatus* interacting with immature DC (iDC) at four time points over 12 h identified 210 differentially regulated genes which showed significant up-regulation of genes involved in fermentation, drug transport, pathogenesis, and response to oxidative stress (Morton et al., 2011). It was interesting that catalases were not up-regulated by the *A. fumigatus* during interaction with moDC, this contrasts with the data from interactions with neutrophils. This occurs because DCs do not acidify the phagolysosome; this is achieved by tightly controlling ROS generation and contributes to antigen preservation (Watts, 2006).

In contrast to the transcription response in murine lungs there was no differential regulation of the gliotoxin gene cluster. However, *Aspf1* was up-regulated as germ tubes emerged after 9 h co-incubation, which corresponded with an increase in *CCL20* expression in the iDC. The allergen Aspf1 is a fungal ribotoxin; a class of RNases that cleave RNA in ribosomes leading to disrupted protein synthesis and apoptosis in target cells (Lacadena et al., 2007). *CCL20* expression had previously been linked to Aspf1 in an experiment where moDC were co-incubated with purified Aspf1 (Ok et al., 2009). CCL20 is associated with allergic, Th2, responses in conditions such as asthma (Reibman et al., 2003). As an allergen Aspf1 interacts with moDC to induce an allergic response, characterized by *CCL20* expression, which is seen in allergic bronchopulmonary aspergillosis (ABPA; Lacadena et al., 2007).

It has been reported that pDC were able to inhibit the growth of *A. fumigatus* hyphae through contact-independent cytotoxicity (Ramirez-Ortiz et al., 2011). This study indicated a close resemblance between the antifungal activities of pDC and NK cells (Bouzani et al., 2011).

## MONOCYTES

Monocytes originate from the bone marrow and, in response to certain immunological cues, such as IA, they migrate via the circulation to the lungs; where they differentiate into DC (Cramer et al., 2011). In mice, Ly6C^hi^ monocytes express the chemokine receptor CCR2 which appears to influence their migration from bone marrow into the circulation (Serbina and Pamer, 2006). In the steady state, circulating CCR2^+^ Ly6C^hi^ cells differentiate into CD 103^+^ DCs while CCR2 Ly6C^lo^ cells differentiate into CD11b^+^ DCs (Jakubzick et al., 2008). However, in experimental pulmonary *Aspergillus* infection CCR2^+^ Ly6C^HI^ cells differentiate into CD11b^+^ DCs. This results in phagocytosis of fungal conidia through recognition of β- D-glucan by Dectin-1. Although monocytes can inhibit fungal growth it is controversial as to whether they can actually kill *Aspergillus*. Monocytes are also APCs, and through the PRR’s TLR, pentraxin 3, and C type lectins, they can trigger adaptive T cell responses.

The transcriptional responses of healthy human donor monocytes to *A. fumigatus* have been the subject of comparable studies. The response of donor monocytes to *A. fumigatus* conidia measured over 1800 genes differentially expressed when compared to unstimulated control monocytes (Cortez et al., 2006), while over 400 genes were expressed solely in response to *A. fumigatus* conidia compared to monocytes stimulated with lipopolysaccharide (LPS; Rodland et al., 2008). In the study of Cortez et al. (2006), over 80% of monocytes had phagocytosed conidia which was comparable to the results for moDC (Morton et al., 2011). The expression of cytokine receptor encoding genes *IL1* and *IL10* progressively increased over the 6 h time course as did the expression of chemokine receptor genes *CXCL2*, *CCL3*, *CCL4*, and *CCL20*. Of genes that encode PRRs *PTX-3* (long pentraxin 3) expression also increased over this time course. Interestingly, there was down-regulation of *TLR1* and no change in expression of either *TLR2* or *TLR4* (Cortez et al., 2006). The study from Rodland et al. (2008) differed in that a significant increase in expression of TLR5 was observed. This had not been identified previously as having a role in host defense against moulds; it was hypothesized that this may suggest a role for regulatory T cells in immunity to IA. By contrast, there was no up-regulation of other *TLR* gene expression; however they did observe up-regulation of IRF8 and IRAK1 expression. Interferon regulating factors are transcription factors involved in pro-inflammatory cytokine responses. Another potentially important finding was production of anti-apoptotic gene responses in *A. fumigatus* infected monocytes. Further *in vitro* experiments by this group support a role for TLR5 in immune response to *A. fumigatus* (Rodland et al., 2011).

*Aspergillus fumigatus* exists as different morphotypes (conidia and hyphae) during infection, the transcriptional response of donor monocytes to *A. fumigatus* hyphae has also been measured (Loeffler et al., 2009). After 3 h co-incubation 602 monocyte genes were differentially regulated in response to *A. fumigatus* hyphae compared to 206 in response to resting conidia. A range of cytokines and chemokines had increased expression in response to *A. fumigatus* conidia and hyphae however there was no differential regulation of either *TLR2* or *TLR4*. In parallel ELISA assays the authors noted increased production of IL-8, CCL2, and CCL20 which together with the finding of increased expression of plasminogen activating genes and *PTX-3* might suggest a mechanism for pulmonary thrombosis and local tissue injury at the site of *Aspergillus* infection.

From the findings of these three transcriptome studies, innate immune molecules, pro-inflammatory cytokines, and immunomodulatory responses appear to be central to the host monocyte response to *Aspergillus* infection. It would be important to translate these data to the clinical setting by experimentally studying the *in vitro* capacity of immunosuppressed patients’ monocytes to respond to *Aspergillus *challenge

## INTERACTIONS WITH NK CELLS

Natural killer cells are innate immune lymphocytes that have been extensively studied due to their ability to kill virus infected and transformed cells without any prior immunization (Caligiuri, 2008). They have an immunoregulatory role, which is fulfilled via the release of several cytokines, predominantly interferon-γ (IFN-γ) as well as chemokines and growth factors (Smyth et al., 2005). Upon infection or inflammation, NK cells migrate from the blood to the lungs, where they become activated (Culley, 2009). During infection by non-viral pathogens regulation of NK cell function is relatively indirect, resulting from signals delivered by accessory cells (Newman and Riley, 2007), but there is growing evidence of a direct interplay between NK cells and pathogenic microbes, including bacteria (Sporri et al., 2006), parasites (Hansen et al., 2007), and yeast (Ma et al., 2004). The interaction between NK cells and *A. fumigatus* is a particularly interesting and relatively unexplored field.

The first demonstration of a role for NK cells in the host defense against IA was achieved in a neutropenic mouse model of IA. The early influx of NK cells into the lungs of mice with IA was linked to an increase in pulmonary CCL2; neutralization of CCL2 impaired the recruitment of NK cells and led to increased mortality (Morrison et al., 2003). A similar experimental model indicated that IFN-γ, produced in the lungs by NK cells, triggers antifungal mechanisms and mediates the protective impact of NK cells (Park et al., 2009). This indicated that NK cell-derived IFN-γ was the mediator of the NK cell protective effect against IA, and excluded the involvement of NK cell cytotoxic proteins (e.g., perforin, granzymes, granulysin). NK cell-derived IFN-γ also increased the capacity of macrophages to inhibit the germination of conidia. These findings suggested that NK cells are a critical component of the innate immune response against IA.

More recent studies on the interaction between purified human NK cells and *A. fumigatus* (Bouzani et al., 2011; Schmidt et al., 2011) have both confirmed the ability of NK cells to mount an effective defense against IA but through contrasting mechanisms of antifungal activity. The debate raised by these contrasting studies has been discussed elsewhere (Bouzani et al., 2012). Schmidt et al. (2011) showed that NK cells with or without interleukin-2 (IL-2) stimulation could kill hyphae but not conidia with perforin acting as the mediator of the cytotoxic mechanism. In contrast, Bouzani et al. (2011) demonstrated a two-step antifungal mechanism, where contact-dependent activation of NK cells by hyphae provoked the release of IFN-γ able to damage the fungus. Second, upon its secretion, the NK cell-derived IFN-γ was capable of acting against hyphae that were not in physical contact with the NK cells. This mechanism was found to be independent of the degranulation NK cells (release of perforin). IFN-γ-mediated antifungal activity of NK cells was consistent with the results of the neutropenic mouse model (Park et al., 2009). The pathway through which IFN-γ attacks *A. fumigatus* remains to be elucidated.

The anti-*Aspergillus* activity of invariant natural killer T (iNKT) has also been studied (Cohen et al., 2011). In an immunocompetent mouse model, it was shown that *A. fumigatus* activated iNKT cells in the presence of CD1d^+^ APC. Instead of lipids it was β-1,3 glucan that induced the release of IL-12 by APCs and thereafter the activation of iNKT cells to secrete IFN-γ.

## VACCINE AND THERAPEUTIC DEVELOPMENT

The strong evidence, provided by the studies mentioned in this review, for the ability of immune cells to control the growth of *A. fumigatus* *in vitro* has suggested the possibility of using innate immune cells as vaccines against IA. The ability of purified *A. fumigatus* antigens to modulate the immunity of iDC (Ok et al., 2009) and the capacity of *Aspergillus*-pulsed DC to drive a Th1 host immune response in mice (Bozza et al., 2003) have indicated the vaccination potential of DC (Roy and Klein, 2012). The transfer of NK cells to immunosuppressed patients to generate a protective response against IA is also possible. To date, existing reports show a protective effect of NK cell transfer to animal hosts with IA (Park et al., 2009). However, the promising evidence is counter-balanced by the limited number of studies, the potential side effects and the unknown characteristics of the candidate treatment group.

The *in vitro* interactions of monocytes and *Aspergillus* with the antifungal drugs voriconazole or lipid formulations of amphotericin B have been investigated. The results suggest that under the experimental conditions used these drugs can promote pro-inflammatory immune responses of monocytes to *A. fumigatus* hyphae (Simitsopoulou et al., 2008, 2011), suggesting a possible role in clearing the fungus *in vivo*. The use of exogenous IFN-γ as an immunological adjunct to antifungal therapy has been found to be effective in individual renal transplant cases with *A. fumigatus* pulmonary infection (Armstrong-James et al., 2010). The likely mechanism is by IFN-γ immunotherapy enhancing the ability of pulmonary phagocytic cells to promote pro-inflammatory responses in *Aspergillus* infection which facilitates clearance of the fungus.

## CONCLUSION

Studies of the interactions between *A. fumigatus* and immune cells have contributed greatly to the understanding of the host–pathogen interaction during aspergillosis (**Figure 1**). These have indicated that under *in vitro* conditions the fungus can survive interactions with macrophages and DC through its ability to adapt to the harsh environmental conditions within the host rather than a defined pathology reliant on specific virulence factors. In identifying the roles of lesser studied immune cells in defense against IA it has been possible to identify novel therapeutic strategies that may eventually ease the burden of IA.

## Conflict of Interest Statement

The authors declare that the research was conducted in the absence of any commercial or financial relationships that could be construed as a potential conflict of interest.

## References

[B1] Agarwal R. Whang D. H. Alvero A. B. Visintin I. Lai Y. Segal E. A. (2007). Macrophage migration inhibitory factor expression in ovarian cancer. *Am. J. Obstet. Gynecol.* 196 348.e341–e345 17403417 10.1016/j.ajog.2006.12.030

[B2] Aimanianda V. Bayry J. Bozza S. Kniemeyer O. Perruccio K. Elluru S. R. (2009). Surface hydrophobin prevents immune recognition of airborne fungal spores. *Nature* 460 1117–1121 19713928 10.1038/nature08264

[B3] Armstrong-James D. Teo I. A. Shrivastava S. Petrou M. A. Taube D. Dorling A. (2010). Exogenous interferon-gamma immunotherapy for invasive fungal infections in kidney transplant patients. *Am. J. Transplant.* 10 1796–1803 20353472 10.1111/j.1600-6143.2010.03094.x

[B4] Bellocchio S. Moretti S. Perruccio K. Fallarino F. Bozza S. Montagnoli, C., et al. (2004). TLRs govern neutrophil activity in aspergillosis. *J. Immunol.* 173 7406–7415 15585866 10.4049/jimmunol.173.12.7406

[B5] Bianchi M. Niemiec M. J. Siler U. Urban C. F. Reichenbach J. (2011). Restoration of anti-*Aspergillus* defense by neutrophil extracellular traps in human chronic granulomatous disease after gene therapy is calprotectin-dependent. *J. Allergy Clin. Immunol.* 127 1243–1252.e1247. 21376380 10.1016/j.jaci.2011.01.021

[B6] Bonnett C. R. Cornish E. J. Harmsen A. G. Burritt J. B. (2006). Early neutrophil recruitment and aggregation in the murine lung inhibit germination of *Aspergillus fumigatus* conidia. *Infect. Immun.* 74 6528–6539 16920786 10.1128/IAI.00909-06 PMC1698102

[B7] Bouzani M. Einsele H. Loeffler J. (2012). Functional analysis is a paramount prerequisite for understanding the *in vitro* interaction of human natural killer cells with *Aspergillus fumigatus*. *J. Infect. Dis.* 205 1025–1026; author reply 1026–1027. 22279122 10.1093/infdis/jir877

[B8] Bouzani M. Ok M. Mccormick A. Ebel F. Kurzai O. Morton C. O. (2011). Human NK cells display important antifungal activity against *Aspergillus fumigatus*, which is directly mediated by IFN-gamma release. *J. Immunol.* 187 1369–1376 21697457 10.4049/jimmunol.1003593

[B9] Bozza S. Gaziano R. Spreca A. Bacci A. Montagnoli C. Di Francesco P. (2002). Dendritic cells transport conidia and hyphae of *Aspergillus fumigatus* from the airways to the draining lymph nodes and initiate disparate Th responses to the fungus. *J. Immunol.* 168 1362–1371 11801677 10.4049/jimmunol.168.3.1362

[B10] Bozza S. Perruccio K. Montagnoli C. Gaziano R. Bellocchio S. Burchielli E. (2003). A dendritic cell vaccine against invasive aspergillosis in allogeneic hematopoietic transplantation. *Blood* 102 3807–3814 12791648 10.1182/blood-2003-03-0748

[B11] Brakhage A. A. Bruns S. Thywissen A. Zipfel P. F. Behnsen J. (2010). Interaction of phagocytes with filamentous fungi. *Curr. Opin. Microbiol.* 13 409–415 20627805 10.1016/j.mib.2010.04.009

[B12] Brown G. D. (2006). Dectin-1: a signalling non-TLR pattern-recognition receptor. *Nat. Rev. Immunol.* 6 33–43 16341139 10.1038/nri1745

[B13] Bruns S. Kniemeyer O. Hasenberg M. Aimanianda V. Nietzsche S. Thywissen A. (2010). Production of extracellular traps against *Aspergillus fumigatus in vitro* and in infected lung tissue is dependent on invading neutrophils and influenced by hydrophobin RodA. *PLoS Pathog.* 6 e1000873 10.1371/journal.ppat.1000873 PMC2861696 20442864

[B14] Burns S. Thrasher A. J. (2004). Dendritic cells: the bare bones of immunity. *Curr. Biol.* 14 R965–R967 15556856 10.1016/j.cub.2004.10.044

[B15] Caligiuri M. A. (2008). Human natural killer cells. *Blood* 112 461–469 18650461 10.1182/blood-2007-09-077438 PMC2481557

[B16] Cohen N. R. Tatituri R. V. Rivera A. Watts G. F. Kim E. Y. Chiba A. (2011). Innate recognition of cell wall beta-glucans drives invariant natural killer T cell responses against fungi. *Cell Host Microbe* 10 437–450 22100160 10.1016/j.chom.2011.09.011 PMC5016029

[B17] Comera C. Andre K. Laffitte J. Collet X. Galtier P. Maridonneau-Parini I. (2007). Gliotoxin from *Aspergillus fumigatus* affects phagocytosis and the organization of the actin cytoskeleton by distinct signalling pathways in human neutrophils. *Microbes Infect.* 9 47–54 17196420 10.1016/j.micinf.2006.10.009

[B18] Cornish E. J. Hurtgen B. J. Mcinnerney K. Burritt N. L. Taylor R. M. Jarvis J. N. (2008). Reduced nicotinamide adenine dinucleotide phosphate oxidase-independent resistance to *Aspergillus fumigatus* in alveolar macrophages. *J. Immunol.* 180 6854–6867 18453606 10.4049/jimmunol.180.10.6854

[B19] Cortez K. J. Lyman C. A. Kottilil S. Kim H. S. Roilides E. Yang J. (2006). Functional genomics of innate host defense molecules in normal human monocytes in response to *Aspergillus fumigatus*. *Infect. Immun.* 74 2353–2365 16552065 10.1128/IAI.74.4.2353-2365.2006 PMC1418921

[B20] Cramer R. A. Rivera A. Hohl T. M. (2011). Immune responses against *Aspergillus fumigatus*: what have we learned? *Curr. Opin. Infect. Dis.* 24 315–322 21666456 10.1097/QCO.0b013e328348b159 PMC3733365

[B21] Culley F. J. (2009). Natural killer cells in infection and inflammation of the lung. *Immunology* 128 151–163 19740372 10.1111/j.1365-2567.2009.03167.x PMC2767305

[B22] De Pauw B. Walsh T. J. Donnelly J. P. Stevens D. A. Edwards J. E. Calandra T. (2008). Revised definitions of invasive fungal disease from the European Organization for Research and Treatment of Cancer/Invasive Fungal Infections Cooperative Group and the National Institute of Allergy and Infectious Diseases Mycoses Study Group (EORTC/MSG) Consensus Group. *Clin. Infect. Dis.* 46 1813–1821 18462102 10.1086/588660 PMC2671227

[B23] Fallon J. P. Reeves E. P. Kavanagh K. (2010). Inhibition of neutrophil function following exposure to the *Aspergillus fumigatus* toxin fumagillin. *J. Med. Microbiol.* 59 625–633 20203215 10.1099/jmm.0.018192-0

[B24] Gugnani H. C. (2003). Ecology and taxonomy of pathogenic aspergilli. *Front. Biosci.* 8 s346–s357 12700046 10.2741/1002

[B25] Hansen D. S. Bernard N. J. Nie C. Q. Schofield L. (2007). NK cells stimulate recruitment of CXCR3+ T cells to the brain during Plasmodium berghei-mediated cerebral malaria. *J. Immunol.* 178 5779–5788 17442962 10.4049/jimmunol.178.9.5779

[B26] Hasenberg M. Behnsen J. Krappmann S. Brakhage A. Gunzer M. (2011). Phagocyte responses towards *Aspergillus fumigatus*. *Int. J. Med. Microbiol.* 301 436–444 21571589 10.1016/j.ijmm.2011.04.012

[B27] Ibrahim-Granet O. Jouvion G. Hohl T. M. Droin-Bergere S. Philippart F. Kim O. Y. (2010). *In vivo* bioluminescence imaging and histopathopathologic analysis reveal distinct roles for resident and recruited immune effector cells in defense against invasive aspergillosis. *BMC Microbiol.* 10 105 10.1186/1471-2180-10-105 PMC2859869 20377900

[B28] Ibrahim-Granet O. Philippe B. Boleti H. Boisvieux-Ulrich E. Grenet D. Stern M. (2003). Phagocytosis and intracellular fate of *Aspergillus fumigatus* conidia in alveolar macrophages. *Infect. Immun.* 71 891–903 12540571 10.1128/IAI.71.2.891-903.2003 PMC145364

[B29] Jakubzick C. Tacke F. Ginhoux F. Wagers A. J. Van Rooijen N. Mack M. (2008). Blood monocyte subsets differentially give rise to CD103+ and CD103- pulmonary dendritic cell populations. *J. Immunol.* 180 3019–3027 18292524 10.4049/jimmunol.180.5.3019

[B30] Lacadena J. Alvarez-Garcia E. Carreras-Sangra N. Herrero-Galan E. Alegre-Cebollada J. Garcia-Ortega L. (2007). Fungal ribotoxins: molecular dissection of a family of natural killers. *FEMS Microbiol. Rev.* 31 212–237 17253975 10.1111/j.1574-6976.2006.00063.x

[B31] Latge J. P. (1999). *Aspergillus fumigatus* and aspergillosis. *Clin. Microbiol. Rev.* 12 310–350 10194462 10.1128/cmr.12.2.310 PMC88920

[B32] Loeffler J. Haddad Z. Bonin M. Romeike N. Mezger M. Schumacher U. (2009). Interaction analyses of human monocytes co-cultured with different forms of *Aspergillus fumigatus*. *J. Med. Microbiol.* 58 49–58 19074652 10.1099/jmm.0.003293-0

[B33] Ma L. L. Wang C. L. Neely G. G. Epelman S. Krensky A. M. Mody C. H. (2004). NK cells use perforin rather than granulysin for anticryptococcal activity. *J. Immunol.* 173 3357–3365 15322199 10.4049/jimmunol.173.5.3357

[B34] McCormick A. Heesemann L. Wagener J. Marcos V. Hartl D. Loeffler J. (2010a). NETs formed by human neutrophils inhibit growth of the pathogenic mold *Aspergillus fumigatus*. *Microbes Infect.* 12 928–936 20603224 10.1016/j.micinf.2010.06.009

[B35] McCormick A. Loeffler J. Ebel F. (2010b). *Aspergillus fumigatus*: contours of an opportunistic human pathogen. *Cell. Microbiol.* 12 1535–1543 20716206 10.1111/j.1462-5822.2010.01517.x

[B36] McDonagh A. Fedorova N. D. Crabtree J. Yu Y. Kim S. Chen D. (2008). Sub-telomere directed gene expression during initiation of invasive aspergillosis. *PLoS Pathog.* 4 e1000154 10.1371/journal.ppat.1000154 PMC2526178 18787699

[B37] Mezger M. Kneitz S. Wozniok I. Kurzai O. Einsele H. Loeffler J. (2008). Proinflammatory response of immature human dendritic cells is mediated by dectin-1 after exposure to *Aspergillus fumigatus* germ tubes. *J. Infect. Dis.* 197 924–931 18279049 10.1086/528694

[B38] Mircescu M. M. Lipuma L. Van Rooijen N. Pamer E. G. Hohl T. M. (2009). Essential role for neutrophils but not alveolar macrophages at early time points following *Aspergillus fumigatus* infection. *J. Infect. Dis.* 200 647–656 19591573 10.1086/600380 PMC2745295

[B39] Morrison B. E. Park S. J. Mooney J. M. Mehrad B. (2003). Chemokine-mediated recruitment of NK cells is a critical host defense mechanism in invasive aspergillosis. *J. Clin. Invest.* 112 1862–1870 14679181 10.1172/JCI18125 PMC296992

[B40] Morton C. O. Varga J. J. Hornbach A. Mezger M. Sennefelder H. Kneitz S. (2011). The temporal dynamics of differential gene expression in *Aspergillus fumigatus* interacting with human immature dendritic cells *in vitro*. *PLoS ONE* 6 e16016 10.1371/journal.pone.0016016 PMC3021540 21264256

[B41] Neofytos D. Horn D. Anaissie E. Steinbach W. Olyaei A. Fishman J. (2009). Epidemiology and outcome of invasive fungal infection in adult hematopoietic stem cell transplant recipients: analysis of Multicenter Prospective Antifungal Therapy (PATH) Alliance registry. *Clin. Infect. Dis.* 48 265–273 19115967 10.1086/595846

[B42] Netea M. G. Warris A. Van Der Meer J. W. Fenton M. J. Verver-Janssen T. J. Jacobs L. E. (2003). *Aspergillus fumigatus* evades immune recognition during germination through loss of toll-like receptor-4-mediated signal transduction. *J. Infect. Dis.* 188 320–326 12854089 10.1086/376456

[B43] Newman K. C. Riley E. M. (2007). Whatever turns you on: accessory-cell-dependent activation of NK cells by pathogens. *Nat. Rev. Immunol.* 7 279–291 17380157 10.1038/nri2057

[B44] O’Gorman C. M. Fuller H. T. Dyer P. S. (2009). Discovery of a sexual cycle in the opportunistic fungal pathogen *Aspergillus fumigatus*. *Nature* 457 471–474 19043401 10.1038/nature07528

[B45] Ok M. Latge J. P. Baeuerlein C. Ebel F. Mezger M. Topp M. (2009). Immune responses of human immature dendritic cells can be modulated by the recombinant *Aspergillus fumigatus* antigen Aspf1. *Clin. Vaccine Immunol.* 16 1485–1492 19675222 10.1128/CVI.00175-08 PMC2756848

[B46] Oppenheim J. J. Tewary P. De La Rosa G. Yang D. (2007). Alarmins initiate host defense. *Adv. Exp. Med. Biol.* 601 185–194 17713005 10.1007/978-0-387-72005-0_19

[B47] Osugi Y. Vuckovic S. Hart D. N. (2002). Myeloid blood CD11c(+) dendritic cells and monocyte-derived dendritic cells differ in their ability to stimulate T lymphocytes. *Blood* 100 2858–2866 12351396 10.1182/blood.V100.8.2858

[B48] Park S. J. Hughes M. A. Burdick M. Strieter R. M. Mehrad B. (2009). Early NK cell-derived IFN-{gamma} is essential to host defense in neutropenic invasive aspergillosis. *J. Immunol.* 182 4306–4312 19299730 10.4049/jimmunol.0803462 PMC3030967

[B49] Park S. J. Mehrad B. (2009). Innate immunity to *Aspergillus* species. *Clin. Microbiol. Rev.* 22 535–551 19822887 10.1128/CMR.00014-09 PMC2772361

[B50] Philippe B. Ibrahim-Granet O. Prevost M. C. Gougerot-Pocidalo M. A. Sanchez Perez M. Van Der Meeren A. (2003). Killing of *Aspergillus fumigatus* by alveolar macrophages is mediated by reactive oxidant intermediates. *Infect. Immun.* 71 3034–3042 12761080 10.1128/IAI.71.6.3034-3042.2003 PMC155721

[B51] Ramirez-Ortiz Z. G. Lee C. K. Wang J. P. Boon L. Specht C. A. Levitz S. M. (2011). A nonredundant role for plasmacytoid dendritic cells in host defense against the human fungal pathogen *Aspergillus fumigatus*. *Cell Host Microbe* 9 415–424 21575912 10.1016/j.chom.2011.04.007 PMC3100664

[B52] Reibman J. Hsu Y. Chen L. C. Bleck B. Gordon T. (2003). Airway epithelial cells release MIP-3alpha/CCL20 in response to cytokines and ambient particulate matter. *Am. J. Respir. Cell Mol. Biol.* 28 648–654 12760962 10.1165/rcmb.2002-0095OC

[B53] Rodland E. K. Ager-Wick E. Halvorsen B. Muller F. Froland S. S. (2011). Toll like receptor 5 (TLR5) may be involved in the immunological response to *Aspergillus fumigatus in vitro*. *Med. Mycol.* 49 375–379 21067314 10.3109/13693786.2010.531772

[B54] Rodland E. K. Mattingsdal M. Olstad O. K. Ovstebo R. Kierulf P. Muller F. (2008). Expression of genes in normal human monocytes in response to *Aspergillus fumigatus*. *Med. Mycol.* 46 327–336 18415839 10.1080/13693780701874507

[B55] Roy R. M. Klein B. S. (2012). Dendritic cells in antifungal immunity and vaccine design. *Cell Host Microbe* 11 436–446 22607797 10.1016/j.chom.2012.04.005 PMC3401965

[B56] Schmidt S. Tramsen L. Hanisch M. Latge J. P. Huenecke S. Koehl U. (2011). Human natural killer cells exhibit direct activity against *Aspergillus fumigatus* hyphae, but not against resting conidia. *J. Infect. Dis.* 203 430–435 21208932 10.1093/infdis/jiq062 PMC3071101

[B57] Schrettl M. Ibrahim-Granet O. Droin S. Huerre M. Latge J. P. Haas H. (2010). The crucial role of the *Aspergillus fumigatus* siderophore system in interaction with alveolar macrophages. *Microbes Infect.* 12 1035–1041 20659583 10.1016/j.micinf.2010.07.005 PMC2977081

[B58] Segal B. H. (2007). Role of macrophages in host defense against aspergillosis and strategies for immune augmentation. *Oncologist* 12 7–13 18039634 10.1634/theoncologist.12-S2-7

[B59] Segers R. Butt T. M. Kerry B. R. Peberdy J. F. (1994). The nematophagous fungus Verticillium chlamydosporium produces a chymoelastase-like protease which hydrolyses host nematode proteins *in situ*. *Microbiology* 140(Pt 10) 2715–2723 8000541 10.1099/00221287-140-10-2715

[B60] Serbina N. V. Cherny M. Shi C. Bleau S. A. Collins N. H. Young J. W. (2009). Distinct responses of human monocyte subsets to *Aspergillus fumigatus* conidia. *J. Immunol.* 183 2678–2687 19635902 10.4049/jimmunol.0803398 PMC2753882

[B61] Serbina N. V. Pamer E. G. (2006). Monocyte emigration from bone marrow during bacterial infection requires signals mediated by chemokine receptor CCR2. *Nat. Immunol.* 7 311–317 16462739 10.1038/ni1309

[B62] Silva M. T. (2011). Macrophage phagocytosis of neutrophils at inflammatory/infectious foci: a cooperative mechanism in the control of infection and infectious inflammation. *J. Leukoc. Biol.* 89 675–683 21169518 10.1189/jlb.0910536

[B63] Simitsopoulou M. Roilides E. Georgiadou E. Paliogianni F. Walsh T. J. (2011). Differential transcriptional profiles induced by amphotericin B formulations on human monocytes during response to hyphae of *Aspergillus fumigatus*. *Med. Mycol.* 49 176–185 20807031 10.3109/13693786.2010.510539

[B64] Simitsopoulou M. Roilides E. Paliogianni F. Likartsis C. Ioannidis J. Kanellou K. (2008). Immunomodulatory effects of voriconazole on monocytes challenged with *Aspergillus fumigatus*: differential role of Toll-like receptors. *Antimicrob. Agents Chemother.* 52 3301–3306 18625774 10.1128/AAC.01018-07 PMC2533507

[B65] Slesiona S. Gressler M. Mihlan M. Zaehle C. Schaller M. Barz D. (2012). Persistence versus escape: *Aspergillus terreus* and *Aspergillus fumigatus* employ different strategies during interactions with macrophages. *PLoS ONE* 7 e31223 10.1371/journal.pone.0031223 PMC3272006 22319619

[B66] Smyth M. J. Cretney E. Kelly J. M. Westwood J. A. Street S. E. Yagita H. (2005). Activation of NK cell cytotoxicity. *Mol. Immunol.* 42 501–510 15607806 10.1016/j.molimm.2004.07.034

[B67] Sporri R. Joller N. Albers U. Hilbi H. Oxenius A. (2006). MyD88-dependent IFN-gamma production by NK cells is key for control of *Legionella pneumophila* infection. *J. Immunol.* 176 6162–6171 16670325 10.4049/jimmunol.176.10.6162

[B68] St Leger R. J. Charnley A. K. Cooper R. M. (1987). Characterization of cuticle-degrading proteases produced by the entomopathogen *Metarhizium anisopliae*. *Arch. Biochem. Biophys.* 253 221–232 3545084 10.1016/0003-9861(87)90655-2

[B69] Sugui J. A. Kim H. S. Zarember K. A. Chang Y. C. Gallin J. I. Nierman W. C. (2008). Genes differentially expressed in conidia and hyphae of *Aspergillus fumigatus* upon exposure to human neutrophils. *PLoS ONE* 3 e2655 10.1371/journal.pone.0002655 PMC2481287 18648542

[B70] Teutschbein J. Albrecht D. Potsch M. Guthke R. Aimanianda V. Clavaud C. (2010). Proteome profiling and functional classification of intracellular proteins from conidia of the human-pathogenic mold *Aspergillus fumigatus*. *J. Proteome Res.* 9 3427–3442 20507060 10.1021/pr9010684

[B71] Thywissen A. Heinekamp T. Dahse H. M. Schmaler-Ripcke J. Nietzsche S. Zipfel P. F. (2011). Conidial dihydroxynaphthalene melanin of the human pathogenic fungus *aspergillus fumigatus* interferes with the host endocytosis pathway. *Front. Microbiol. *2:96. 10.3389/fmicb.2011.00096 PMC3128974 21747802

[B72] Volling K. Brakhage A. A. Saluz H. P. (2007). Apoptosis inhibition of alveolar macrophages upon interaction with conidia of *Aspergillus fumigatus*. *FEMS Microbiol. Lett.* 275 250–254 17714483 10.1111/j.1574-6968.2007.00883.x

[B73] Watts C. (2006). Phagosome neutrality in host defense. *Cell* 126 17–19 16839868 10.1016/j.cell.2006.06.031

[B74] Wuthrich M. Deepe G. S. Jr. Klein B. (2012). Adaptive immunity to fungi. *Ann. Rev. Immunol.* 30 115–148 22224780 10.1146/annurev-immunol-020711-074958 PMC3584681

